# Integrated Multi-Omics Analyses Identify Core Determinants Governing Newcastle Disease Virus Oncolytic Therapy Efficacy Under Tumor Heterogeneity

**DOI:** 10.3390/microorganisms14102156

**Published:** 2026-09-24

**Authors:** Shufeng Feng, Yongxin Zhu, Lu Cui, Hongchun Yang, Huimin Duan, Xuefeng Li, Hai Li, Changbo Ou

**Affiliations:** 1 College of Animal Science and Technology, Guangxi University, Nanning 530004, China; feng_xjtu0204@163.com (S.F.); yanghc0211@163.com (H.Y.); 2 School of Basic Medical Sciences, Xi’an Jiaotong University Health Science Center, Xi’an Jiaotong University, Xi’an 710061, China; zyx2023@stu.xjtu.edu.cn (Y.Z.); duanhum@stu.xjtu.edu.cn (H.D.); lixuefeng@xjtu.edu.cn (X.L.); 3 National & Local Joint Engineering Research Center of Biodiagnosis and Biotherapy, The Second Affiliated Hospital of Xi’an Jiaotong University, Xi’an 710004, China

**Keywords:** Newcastle disease virus, oncolytic virotherapy, multi-omics analysis, host factors, antiviral defense

## Abstract

Newcastle disease virus (NDV) is a promising oncolytic virus, but tumor heterogeneity constrains its clinical translation. The key determinants driving variable NDV oncolytic potency amid tumor heterogeneity remain incompletely elucidated. The main scope of this study included analyzing 22 human cancer cell lines representing 11 tumor types and identifying five gene co-expression modules associated with NDV susceptibility through weighted gene co-expression network analysis. Integrative multi-omics analyses of NDV-infected HCT116 cells, the least-susceptible cell line in our panel, predicted metabolic processes, innate immune defenses, and multiple forms of cell death as candidate pathways modulating NDV oncolytic efficacy. These key bioinformatic findings were further validated by genome-wide CRISPR-Cas9 screening, which uncovered *POLR2G* and *SREBF1* as central components of the pro-oncolytic network and *TNFRSF1A*, *TNFAIP3*, and *CASP9* as core mediators of the anti-oncolytic network. Their functional roles were further verified via genetic manipulation and pharmacological perturbation of JAK-STAT, TLR4, and apoptotic signaling. In conclusion, our findings support a bidirectional model wherein host metabolic programs promote NDV replication and oncolysis, whereas antiviral-innate immune signaling restricts them, providing candidate targets for host-directed combination therapy amid tumor heterogeneity.

## 1. Introduction

Cancer has become one of the most important public health issues in the world, and its incidence and mortality rates continue to rise [1]. According to global cancer statistics released by the International Agency for Research on Cancer (IARC), there were approximately 20 million new cancer cases and 9.7 million cancer-related deaths worldwide in 2022 [2]. It is estimated that, by 2050, the number of new cases will increase to 35 million, an increase of approximately 77% compared to 2022 [3]. Although comprehensive treatment strategies such as surgery, radiotherapy, chemotherapy, targeted therapy and immunotherapy have been continuously developed in recent years, the treatment effect is still limited for patients with advanced or metastatic tumors, and the recurrence rate and mortality rate of patients remain high [4,5,6,7,8]. Therefore, developing safer and more efficient new anti-tumor treatment strategies is still an important direction for current tumor research. Oncolytic viruses (OVs) are an emerging antitumor therapeutic strategy that can selectively infect, replicate in and lyse tumor cells, while activating the body’s anti-tumor immune response [9]. In recent years, with the development of viral genetic engineering and reverse genetics technology, a variety of natural or genetically engineered oncolytic viruses have entered the clinical research stage [10]. Currently, modified adenovirus type 5 and genetically engineered herpes simplex virus type 1 (HSV-1) have been successfully applied in clinical practice [11]. Among them, Talimogene laherparepvec (T-VEC), derived from HSV-1, has been approved for the treatment of melanoma, confirming the clinical value of oncolytic viruses in the treatment of solid tumors [12]. More recently, the US Food and Drug Administration granted accelerated approval to TUDRIQEV (vusolimogene oderparepvec-wtpg; formerly RP1), in combination with nivolumab, for adult patients with unresectable advanced cutaneous melanoma that progressed following anti-PD-1–based therapy. In addition, there are currently about 180 clinical trials of oncolytic viruses involving DNA and RNA viruses underway worldwide, further promoting the development of oncolytic virus therapy [13]. However, due to factors such as the limited spread of viruses within solid tumors, the tumor’s immunosuppressive microenvironment, and the host’s natural antiviral response, the therapeutic effect of existing oncolytic viruses in most solid tumors still has considerable room for improvement [14].

Newcastle disease virus (NDV) is a single-stranded negative-sense RNA virus and is one of the natural oncolytic viruses that has received much attention in recent years [15]. Compared with some DNA oncolytic viruses, NDV has the characteristics of natural tumor selectivity, does not integrate into the host genome, and has extremely low replication ability in normal human tissues, thus having high biosafety [16]. Numerous studies have shown that NDV can preferentially infect tumor cells deficient in interferon (IFN) signaling, directly lyse tumor cells through viral replication, and induce immunogenic cell death (ICD), promoting the release of type I interferon, inflammatory factors, and chemokines, further activating the body’s innate and adaptive anti-tumor immune responses [17,18,19]. According to recent reports, the Phase I clinical study of NDV-GT further demonstrated the favorable safety profile, tumor specificity, and tumor-targeting potential of NDV, as well as the feasibility of repeated intravenous administration, thereby providing important evidence supporting its clinical application [20].

Colorectal cancer is one of the malignant tumors with the highest incidence and mortality rates worldwide, and it is also a solid tumor model in which NDV is widely studied [21]. Studies have shown that NDV can exert its anti-colorectal cancer effect through multiple mechanisms such as inducing oxidative stress, mitochondrial dysfunction, apoptosis, and immune activation [22]. However, NDV treatment of colorectal cancer and other solid tumors still faces many challenges, including insufficient viral replication efficiency in tumor tissues, rapid clearance of the virus by host innate antiviral immunity, and significant differences in the sensitivity of different tumor cells to NDV [23]. Increasing research suggests that intrinsic molecular characteristics of host cells are crucial determinants of viral replication efficiency and oncolytic efficacy [24]. However, key host factors regulating NDV infection and oncolytic activity remain poorly elucidated, particularly lacking global screening studies based on multiple tumor models.

Here, using 22 human tumor cell lines derived from 11 distinct tissue types as a model recapitulating the heterogeneous tumor microenvironment, we conducted integrated bioinformatic analyses coupled with genetic manipulation and pharmacological perturbation to dissect host determinants governing variable NDV oncolytic potency amid tumor heterogeneity and identify potential targets for host-directed combination therapy.

## 2. Materials and Methods

### 2.1. Cells, Plasmids, and Virus Strain

All human cell lines in Table 1 were cultured at 37 °C with 5% (*v*/*v*) CO_2_. BxPC3, KYSE-150, KYSE30, and SW620 were cultured in RPMI 1640 supplemented with 10% fetal bovine serum and 1% penicillin/streptomycin. The other cells were cultured in DMEM supplemented with 10% fetal bovine serum and 1% penicillin/streptomycin.

Human CRISPR Knockout Pooled Library (GeCKO v2) (Pooled Libraries #1000000048) and plasmid lentiCRISPR v2 (Plasmid #52961), pCMV-VSV-G (Plasmid #8454), *p*U6-BbsI_CBh-Cas9-T2A-mCherry (Plasmid #64324), pMD2.G (Plasmid #12259) and psPAX2 (Plasmid #12260) were purchased from Addgene (Watertown, MA, USA).

NDV LaSota (GenBank: AY845400) strains were previously maintained in our laboratory.

### 2.2. LDH Release Assay

Cells were seeded in 96-well plates at a density of 1.0 × 10^4^ cells/well and allowed to adhere for 24 h. Following attachment, cells were treated with serum-free medium containing experimental compounds, while untreated cells served as controls. Plates were incubated at 37 °C for specified durations with or without infection of NDV. Cells were pretreated with the specific inhibitors, Ruxolitinib (10 μM, MCE, HY-50856, Monmouth Junction, NJ, USA), Resatorvid (0.5 μM, MCE, HY-11109, Monmouth Junction, NJ, USA), BAY 11-7082 (1 μM, MCE, HY-13453, Monmouth Junction, NJ, USA), Z-LEHD-FMK TFA (1 μM, Selleck, S7313, Houston, TX, USA) and Z-VAD-FMK (10 μM, MCE, HY-16658B, Monmouth Junction, NJ, USA), for 6 h prior to NDV infection. LDH release was measured according to the manufacturer’s instructions (Beyotime, C0017, Shanghai, China). The LDH release reagent was added to the pre-prepared maximum-release control to induce complete cell lysis. Then, the prepared LDH working solution was added to the 96-well plate, and the plate was incubated at room temperature for 30 min with gentle shaking while wrapped in foil. Absorbance was measured at 490 nm using a microplate reader (Thermo Fisher Scientific, Waltham, MA, USA).

### 2.3. Weighted Gene Co-Expression Network Analysis (WGCNA)

R (v4.2.0) package WGCNA was employed to identify key modular genes and assess their relevance to oncolytic efficiency at 12 h and 24 h post-infection. Initially, 22 cell lines’ baseline transcriptomic profiles were obtained from the Cancer Cell Line Encyclopedia through the Broad Institute DepMap portal. The WGCNA input consisted of the FPKM-normalized expression profiles for the same 22 cell lines. Gene identifiers were mapped to HGNC symbols, and the 10,000 genes with the largest standard deviations across the cell-line panel were retained to reduce low-variance background before network construction. Next, the pick Soft Threshold function was utilized to select the appropriate soft threshold β and generate the topological overlap matrix (TOM). To generate the hierarchical clustering tree for module identification, the minimal module size was set as 20 and the mergeCutHeight parameter set as 0.25. Following module identification, the cluster most significantly associated with oncolytic efficiency was selected based on hierarchical clustering analysis. Transcriptomic profiles of the 22 cancer cell lines were obtained from Cancer Cell Line Encyclopedia (CCLE) (Datasets | Cancer Cell Line Encyclopedia (CCLE)). The expression matrix was normalized using FPKM. Gene identifiers were converted to HGNC gene symbols using org.Hs.eg.db.

### 2.4. CRISPR-Cas9 Knockout Screening

Genome-wide CRISPR–Cas9 knockout (KO) screening was conducted using a lentiviral sgRNA library containing 123,411 single-guide RNAs (sgRNAs) targeting 19,050 protein-coding genes in the human genome [25]. After lentiviral packaging, HCT116 cells were infected with the lentiviral sgRNA library in the presence of 8 μg/mL polybrene. At 12 h post-infection, the medium was replaced with fresh culture medium containing 5% FBS, and the cells were cultured for another 12 h. Puromycin (1.5 μg/mL) was then added for antibiotic selection. Control cells were cultured in the same puromycin-containing medium. After all control cells died, the surviving infected cells were collected and expanded for subsequent experiments. On day 7 after selection, cells were infected with NDV at an MOI of 5 for 48 h, while an uninfected population was maintained as the negative control. Genomic DNA was subsequently isolated, and changes in sgRNA abundance between NDV-infected and control samples were quantified by next-generation sequencing.

The surviving cells were infected with NDV at an MOI of 5. At 48 h post-infection, the surviving cells were harvested, and genomic DNA was extracted using a genomic DNA extraction kit according to the manufacturer’s instructions. Integrated sgRNAs were PCR-amplified (Adaptor_F: AATGGACTATCATATGCTTACCGTAACTTGAAAGTATTTCG; Adaptor_R: TCTACTATTCTTTCCCCTGCACTGTTGTGGGCGATGTGCGCTCTG), gel-purified, and prepared for sequencing. Final library sequencing was performed on the Illumina HiSeq 2500 platform to identify genes modulating NDV-mediated cytotoxicity.

### 2.5. High-Throughput Data Analysis

Sequencing data from the CRISPR–Cas9 knockout screen were analyzed using R (v4.2.0) with the edgeR package (v3.40.2). The count matrix generated by *processAmplicons* was normalized using *calcNormFactors*. Differential sgRNA abundance between NDV-infected and control groups was determined using *exactTest*. Gene-level enrichment analysis was performed by aggregating sgRNA signals for each gene using the *camera* gene-set test [26].

### 2.6. RNA Sequencing

HCT116 cells were infected with NDV at an MOI of 5, while untreated cells were used as the control group. At 24 h post-infection, total RNA was extracted from cells for transcriptome analysis by RNA sequencing (RNA-seq). RNA isolation was performed using the RNeasy Plus Mini Kit (Qiagen, 74314, Hilden, Westphalia, Germany) according to the manufacturer’s protocol. The RNA integrity number (RIN) values ranged from 8.4 to 9.8, indicating high RNA quality suitable for sequencing. Sequencing libraries were constructed using the Illumina library preparation protocol (Illumina Inc., San Diego, CA, USA). The libraries were subsequently sequenced on an Illumina HiSeq 2500 platform. On average, approximately 56.48 million raw reads were generated per sample, with a mean clean read rate of 96.4%. The average Q30 scores for raw and clean bases were 92.7% and 93.04%, respectively. RNA-seq raw data were uploaded to the National Center for Biotechnology Information database under the accession number GSE297244.

### 2.7. Functional Enrichment Analysis

Gene Ontology (GO) enrichment analysis, including Biological Process (BP), Cellular Component (CC), and Molecular Function (MF), and Kyoto Encyclopedia of Genes and Genomes (KEGG) pathway enrichment analysis were executed using oebiotech (https://metascape.org/gp/#/main/step1, accessed on 13 March 2026). *p* < 0.05 was the cutoff.

### 2.8. Protein–Protein Interaction (PPI) Network Analysis

The common genes identified by WGCNA, CRISPR, and RNA Sequencing were uploaded to the STRING database (https://cn.string-db.org/, accessed on 20 March 2026) to identify interactions between proteins of interest with confidence (≥0.4). The PPI network was imported into Cytoscape software (v3.10.2) for visualization and analyses.

### 2.9. Generation of Stable Cell Lines

To generate *POLR2G*-knockout, *SREBF1*-knockout, *TNFRSF1A*-knockout, *TNFAIP3*-knockout and *CASP9*-knockout stable cell lines, two of the six single guide RNAs (sgRNAs) targeting distinct *POLR2G*, *SREBF1*, *TNFRSF1A*, *TNFAIP3* and *CASP9* genomic sites were selected and the sequence is shown in Table 2. They were respectively cloned into the BbsI-linearized pU6-BbsI_CBh-Cas9-T2A-mCherry using T4 DNA Ligase.

Subsequently, cells were transfected with the constructed plasmids using Lipofectamine 3000 transfection reagent (Invitrogen, L3000015, Waltham, MA, USA) for 72 h. The transfection efficiency was evaluated by quantifying the percentage of mCherry fluorescence. Cells with red fluorescence were sorted with a sorting flow cytometer (FACSAria, BD Biosciences, Ashland, OR, USA), and then knockout cell lines were constructed for each target gene.

To generate a stable cell line with POLR2G overexpression, genomic DNA was extracted from HCT116. *POLR2G* was amplified using ApexHF HS DNA Polymerase FL (Accurate Biology, AG12207, Changsha, China) with primers: CGGCGCCTACTCTAGATGTTCTACCATATCTCCCTAGAGCACGGAAATCCTGCTGC (*p*CDH-EF1-IRES-Puro-*POLR2G*-F) and CCGATTTAAATTCGAATTTCAGCTTACAAGCCCAAGTAATCGTCCATCAGGGAGCC (*p*CDH-EF1-IRES-Puro-*POLR2G*-R). The PCR product was cloned into the *XbaI*-linearized *p*CDH-EF1-IRES-Puro (NovoPro, V017143, Shanghai, China) using the ClonExpress Ultra One Step Cloning Kit (Vazyme, C115, Nanjing, China) per the manufacturer’s instructions. To generate lentiviral particles, 293T cells were co-transfected with *p*CDH-EF1-IRES-Puro-POLR2G, psPAX2, and pMD2.G plasmids using PEI transfection reagent (Merck, 919012, Rahway, NJ, USA) according to the manufacturer’s instructions. Viral supernatant was harvested at 48 h and 72 h post-transfection, filtered (0.45 µm), and pooled. HCT116 cells were transduced with filtered lentivirus in the presence of 8 µg/mL polybrene (Merck, TR-1003, Rahway, NJ, USA) for 48 h. Transduced cells were screened with 1.5 µg/mL puromycin.

### 2.10. Absolute Quantitative PCR (qPCR)

To detect viral genome replication, viral RNA was extracted using the Mini BEST Viral RNA/DNA Extraction Kit Ver 5.0 (TakaRa, 9766, Kyoto, Japan) according to the manufacturer’s protocol. Subsequent absolute quantitative PCR (qPCR) detection was performed using ABclonal 2X Universal SYBR Green Fast qPCR Mix (ABclonal, RK21203, Wuhan, China) with an applied biosystem (Bioer Technology, Hangzhou, China) according to the manufacturer’s protocol. The following absolute qPCR primers targeting the fragment located between M and F genes of NDV were designed according to the complete genome of NDV LaSota strain: F: 5′-CTGCGTCTCTGAGATTGCGC-3′, R: 5′-CTTGCACCTGGAGGGCG-3′. The standards of absolute qPCR were prepared by cloning the PCR products into the pMD^TM^18-T Vector (TakaRa, 6011, Kyoto, Japan) according to the manufacturer’s instructions.

### 2.11. Real-Time Quantitative PCR

Total RNA was extracted from cells with cell lysis buffer (TIANGEN, DP419, Beijing, China) and reverse transcribed into cDNA with ABScript III RT Master Mix for qPCR (ABclonal, RK20428, Wuhan, China). The mRNA expression of related genes was measured using an ABclonal 2X Universal SYBR Green Fast qPCR Mix (ABclonal, RK21203, Wuhan, China) with an applied biosystem (Bioer Technology, Hangzhou, China) according to the manufacturer’s protocol. The data were evaluated via the 2^−△△Ct^ technique and adjusted to the level of *GAPDH* expression. The primer sequences are provided in Table 3.

### 2.12. Western Blotting

For Western blotting, cells were lysed in cold lysis buffer (Beyotime, P0013, Shanghai, China) with 1mM PMSF (Beyotime, ST506, Shanghai, China) and 1× Phosphatase Inhibitor Cocktail A (Beyotime, P1082, Shanghai, China) at 4 °C for 30 min. Lysates were heat-denatured at 100 °C for 15 min in SDS loading buffer, followed by SDS-PAGE electrophoresis and electro-transfer printing to a PVDF membrane (Millipore, ISEQ00010, Burlington, MA, USA). The membrane was blocked with 5% non-fat milk in PBS containing 0.1% Tween-20 (PBST) at room temperature for 2 h and further rinsed with PBST 3 times. Then the membrane was incubated with each of the following primary antibodies overnight at 4 °C, followed by washing 3 times with PBST: anti-GAPDH (1:10,000, Proteintech, Cat No. 60004-1-Ig, Wuhan, China), anti-POLR2G (1:1000, Proteintech, Cat No. 18497-1-AP, Wuhan, China), anti-SREBF1 (1:1000, Proteintech, Cat No. 14088-1-AP, Wuhan, China), anti-TNFRSF1A (1:1000, Proteintech, Cat No. 21574-1-AP, Wuhan, China), anti-TNFAIP3 (1:1000, Proteintech, Cat No. 66695-1-Ig, Wuhan, China), anti-CASP9 (1:1000, Proteintech, Cat No. 10380-1-AP, Wuhan, China). Next, Goat anti-Mouse IgG (H + L) Secondary Antibody-HRP (1:10,000, Thermo Fisher Scientific, 31430, Waltham, MA, USA) or Goat anti-Rabbit IgG (H + L) Secondary Antibody-HRP (1:10,000, Thermo Fisher Scientific, 31460, Waltham, MA, USA) was added and incubated for 45 min at room temperature. After washing 3 times with PBST, the membrane was detected using the Western ECL (Epizyme Biotech, SQ203, Cambridge, MA, USA) substrate and imaged with the Chemi image analysis system (ENFAN-BIAS2600, Shaanxi Enfan Instrument Manufacturing Co., Ltd., Xianyang, China).

### 2.13. Statistical Analysis

Statistical analyses were performed using GraphPad Prism version 8.0 and R version 4.2.0. Data are presented as mean ± SD unless otherwise indicated. For comparisons between two groups, an unpaired two-tailed Student’s *t*-test was used when appropriate. For comparisons involving multiple groups, one-way ANOVA followed by the appropriate post hoc multiple-comparison test was performed. The statistical test used for each experiment is specified in the corresponding figure legend. A *p* < 0.05 was considered statistically significant.

## 3. Results

### 3.1. WGCNA Analysis of Gene Modules Related to the Oncolytic Efficacy of NDV

To systematically characterize the susceptibility of different human cancer cells to NDV-mediated oncolysis, a panel of 22 human cancer cell lines representing 11 tumor types, including brain, esophagus, breast, lung, liver, stomach, pancreas, colorectum, cervix, ovary, and prostate, was established (Figure 1A). Cells were infected with NDV LaSota at a multiplicity of infection (MOI) of 5, and cytotoxicity was quantified by LDH release assays at 12 and 24 hpi (Figure 1B). At 12 hpi, NDV induced only limited cytotoxicity across all tested cell lines, with cell death remaining below 8%. Cytotoxicity increased substantially at 24 hpi; however, most cell lines still exhibited relatively modest killing efficiencies ranging between 10% and 30%. Notably, the colorectal cancer cell line HCT116 displayed the lowest susceptibility to NDV-induced cell death, with only approximately 10% cytotoxicity, indicating that HCT116 is highly resistant to NDV-mediated oncolysis under these experimental conditions (Figure 1B).

To identify host transcriptional programs associated with NDV-mediated cytotoxicity, WGCNA was performed using publicly available transcriptomic profiles from the 22 cancer cell lines. To improve network robustness and reduce background noise, the 10,000 genes with the highest expression variance were selected for network construction. Scale-free topology analysis identified β = 20 as the optimal soft-thresholding power, yielding a scale-free topology fitting index (R^2^ = 0.805), indicating that the constructed network closely approximated the characteristics of biological co-expression networks (Figure 1C,D). Dynamic tree cutting identified 167 co-expression modules (Figure 1E). To simplify downstream analyses, module eigengenes were further subjected to hierarchical clustering using Ward’s minimum variance method, resulting in five major meta-modules representing distinct gene expression patterns across the 22 tumor cell lines (Figure 1F). Correlation analyses between each meta-module and NDV-induced cytotoxicity revealed that the first meta-module exhibited the strongest positive association with NDV-mediated cell killing, whereas the fourth meta-module showed the strongest negative association. The red and blue modules comprised 1380 and 853 genes, respectively (Table S1), representing the principal transcriptional programs positively or negatively associated with NDV susceptibility. These two modules were subsequently selected for functional enrichment analyses and the identification of candidate host factors regulating NDV oncolytic activity.

### 3.2. GO and KEGG Functional Enrichment Analysis and PPI Network Construction of Positively Regulated Genes

To characterize the transcriptional features of the positively correlated gene network, the expression profiles of the 1380 genes within this module were visualized across the 22 cancer cell lines ordered according to NDV-induced cytotoxicity at 12 hpi (Figure 2A). At 12 hpi, clear differences in NDV-induced cytotoxicity were already evident among the 22 cancer cell lines: susceptible cells had begun to die, whereas resistant cells remained largely unaffected. Therefore, this time point was used to relate transcriptional characteristics to differences in NDV susceptibility. Cell lines were arranged from the highest to the lowest level of NDV-mediated cytotoxicity. Consistent with the module–phenotype association identified by WGCNA, genes within this module exhibited progressively higher expression levels in cell lines with greater susceptibility to NDV-induced cytotoxicity, whereas relatively resistant cell lines displayed generally lower expression of these genes (Figure 2A).

To investigate the biological functions represented by this module, Gene Ontology (GO) enrichment analysis was performed using Metascape. Significant enrichment was observed across the three GO categories (Table S2), including biological process (BP), cellular component (CC), and molecular function (MF) (Figure 2B). In the BP category, genes were primarily enriched in cytoplasmic translation, DNA metabolic processes, mitotic cell cycle, ribosome biogenesis, mitochondrial organization, and p53-mediated signal transduction. In the CC category, enriched terms were predominantly associated with the ribosome, mitochondrial matrix, mitochondrial inner membrane, respiratory chain complex, chromosome, and microtubule-organizing center. MF analysis further demonstrated significant enrichment in structural constituents of ribosomes, catalytic activities acting on nucleic acids, DNA endonuclease activity, rRNA binding, oxidoreductase activity, and protein serine/threonine kinase activity, indicating that this module is closely associated with protein synthesis, mitochondrial function, nucleic acid metabolism, and cell cycle regulation.

To further define the signaling pathways represented by the positive regulatory module, KEGG pathway enrichment analysis was performed (Figure 2C) (Table S3). The enriched pathways were mainly clustered into four functional categories, including metabolism, genetic information processing, environmental information processing, and cellular processes. Representative pathways included oxidative phosphorylation, amino acid metabolism, DNA replication, base excision repair, mTOR, AMPK, HIF-1 signaling, autophagy, and peroxisome, highlighting coordinated regulation of mitochondrial metabolism, nutrient-sensing pathways, genome maintenance, and cellular stress responses. Disease-associated pathways, including Parkinson disease, non-alcoholic fatty liver disease, diabetic cardiomyopathy, insulin resistance, and glioma, were also significantly enriched, reflecting the extensive involvement of these genes in cellular metabolic homeostasis and disease-related biological processes.

To identify key functional hubs within the positive regulatory module, a PPI network was constructed using STRING, and the top 100 hub genes were extracted for further analysis. In the resulting network, node color was scaled according to degree centrality, with red indicating a greater node degree and yellow indicating a relatively smaller node degree. These hub genes were subsequently grouped according to their corresponding KEGG functional categories (Figure 2D). The largest cluster was associated with genetic information processing and consisted predominantly of ribosomal proteins (*RPS* and *RPL* family members), mitochondrial ribosomal proteins (*MRPL* family), translation initiation and elongation factors (*EIFs* and *EEF2*), RNA polymerase subunits (*POLR2H* and *POLR2K*), chromatin regulators (*EP300*, *HDAC2*, and *SMARCA4*), and DNA replication or cell cycle-related proteins including *MCM7*, *CHEK2*, *RECQL4*, and *CDK1*, suggesting that protein translation, transcription, and genome maintenance constitute the core functional network of this module. Additional subnetworks corresponded to metabolism, which was enriched for oxidative phosphorylation-related proteins including *ATP5F1D*, *COX5B*, *NDUFB7*, *NDUFS7*, *CYC1*, and *NDUFA13*; environmental information processing, represented by *AKT1*, *NRAS*, and *MAPK3*; cellular processes, containing ubiquitin–proteasome pathway components including *UBB*, *SEC11C*, *PSMC1*, *PSMC5*, and *RAD21*; and human diseases, represented by *RSAD1* and *SECISBP2*. These findings demonstrate that the positively regulated module is organized into several highly interconnected functional subnetworks centered on mitochondrial metabolism, genetic information processing, intracellular signaling, and protein homeostasis.

Collectively, these enrichment analyses indicate that genes within the positive regulatory module form an integrated functional network primarily involved in mitochondrial energy metabolism, protein synthesis, genome stability, and intracellular signaling. The identification of highly connected hub genes across distinct KEGG functional categories further suggests that coordinated regulation of these biological processes contributes to enhanced susceptibility of tumor cells to NDV-mediated oncolysis.

### 3.3. GO and KEGG Functional Enrichment Analysis and PPI Network Construction of Negatively Regulated Genes

To characterize the transcriptional features of the negative regulatory module, the expression profiles of the 853 genes assigned to this module were visualized across the 22 cancer cell lines ranked according to NDV-induced cytotoxicity at 12 hpi (Figure 3A). Consistent with the negative module–phenotype correlation identified by WGCNA, genes within this module exhibited progressively lower expression levels in cell lines with higher susceptibility to NDV-mediated cytotoxicity, whereas relatively resistant cell lines displayed generally higher expression of these genes (Figure 3A), indicating an inverse association between module expression and NDV-induced cytotoxicity.

To investigate the biological functions represented by this module, GO enrichment analysis was performed using Metascape (Figure 3B). Across the three GO categories (Table S2), genes within the negative regulatory module were predominantly enriched in processes related to cell adhesion, actin cytoskeleton organization, regulation of cell projection organization, vascular development, wound response, and receptor-mediated signal transduction. Enriched cellular components included the actin cytoskeleton, cell–substrate junctions, focal adhesions, lamellipodia, podosomes, Golgi membrane, and apical cell structures, whereas molecular function analysis highlighted enrichment for cell adhesion molecule binding, actin binding, integrin binding, kinase binding, phospholipid binding, and protein kinase activity. These findings indicate that the negative regulatory module is primarily associated with cytoskeletal organization, cell adhesion, and intracellular signaling.

KEGG pathway enrichment analysis further demonstrated that genes in this module were significantly enriched in pathways involved in environmental information processing, cellular processes, organismal systems, and human diseases (Figure 3C) (Table S3). Representative signaling pathways included PI3K–Akt, NF-κB, TNF, JAK–STAT, Ras, Hippo, TGF-β, and cytokine–cytokine receptor interaction, together with pathways associated with ECM–receptor interaction, focal adhesion, adherens junction, apoptosis, necroptosis, phagosome, and cytosolic DNA-sensing. In addition, multiple immune- and disease-related pathways, including natural killer cell-mediated cytotoxicity, complement and coagulation cascades, toxoplasmosis, and pathways in cancer, were significantly enriched, suggesting extensive involvement of this module in cell signaling, immune regulation, and host responses to extracellular stimuli.

To identify key hub proteins within the negative regulatory module, a PPI network was constructed using STRING (Figure 3D). In the resulting network, node color was scaled according to degree centrality, with red indicating higher connectivity and yellow indicating relatively lower connectivity. The resulting network was highly interconnected and centered on multiple signaling molecules, including *EGFR*, *MET*, *TGFB1*, *SMAD3*, *CTNNB1*, *FYN*, *PRKCA*, *PRKACB*, *BRCA1*, *SQSTM1*, *UBC*, *PPARG*, *CAV1*, and *NFE2L2*. These hub proteins are functionally linked to receptor tyrosine kinase signaling, TGF-β signaling, cytoskeletal remodeling, protein ubiquitination, oxidative stress responses, and intracellular signal transduction, forming a densely connected regulatory network. Collectively, these results indicate that the negative regulatory module is enriched for genes involved in cell adhesion, cytoskeletal dynamics, signal transduction, and immune-associated pathways.

### 3.4. Integrated Analysis Reveals the Host Regulatory Network Governing NDV-Mediated Oncolysis

Based on the high degree of consistency between the WGCNA modules and RNA-seq responses, genes residing within these overlapping pathways were prioritized as candidate host factors for subsequent screening and experimental validation. Given that HCT116 exhibited the lowest susceptibility to NDV-mediated cytotoxicity among the 22 tumor cell lines (Figure 1B), this cell line was selected as the representative resistant model for subsequent mechanistic investigations. HCT116 cells were infected with NDV LaSota, and RNA sequencing was performed at 24 hpi to characterize host transcriptional responses (Figure 4A). HCT116 was the least susceptible cell line and exhibited a stable cytotoxic response only at approximately 24 hpi; therefore, 24 hpi was selected for RNA-seq analysis.

Differential expression analysis identified 2117 significantly upregulated genes and 752 significantly downregulated genes following NDV infection (Figure 4B) (Table S4). GO enrichment analysis was subsequently performed (Table S5). Upregulated genes were predominantly enriched in biological processes associated with type I/II interferon responses, NF-κB signaling, antigen processing and presentation, pattern recognition receptor signaling, innate antiviral immunity, inflammatory responses, and immune cell activation. In contrast, downregulated genes were mainly associated with ribosome biogenesis, rRNA processing, protein translation, nucleic acid metabolism, DNA replication, cell proliferation, and chromatin organization.

KEGG pathway enrichment analysis further supported these findings (Table S6). Upregulated genes were significantly enriched in multiple immune- and virus-related signaling pathways, including JAK–STAT signaling, NF-κB signaling, RIG-I-like receptor signaling, Toll-like receptor signaling, cytokine–cytokine receptor interaction, and apoptosis-related pathways. Conversely, downregulated genes were primarily enriched in metabolic pathways, DNA replication, cell cycle regulation, and other fundamental cellular biosynthetic processes.

To further identify biological pathways associated with NDV susceptibility, KEGG pathways enriched in the WGCNA-derived positive and negative regulatory modules were intersected with those enriched among RNA-seq upregulated and downregulated genes, respectively (Figure 4C). Fifteen pathways overlapped between the positive regulatory module and downregulated genes, including pathways involved in amino acid metabolism, carbon metabolism, cofactor biosynthesis, glyoxylate metabolism, DNA replication, base excision repair, Fanconi anemia pathway, RNA polymerase, and HIF-1 signaling, indicating that genes positively associated with NDV-mediated cytotoxicity were broadly suppressed following NDV infection.

In contrast, 32 pathways were shared between the negative regulatory module and upregulated genes. These overlapping pathways were mainly clustered into four functional categories: immune and inflammatory signaling, cell death regulation, innate immune defense, and pathogen infection-related pathways. Representative pathways included JAK–STAT, NF-κB, TNF, cytokine–cytokine receptor interaction, apoptosis, necroptosis, cytosolic DNA-sensing, NOD-like receptor signaling, natural killer cell-mediated cytotoxicity, complement and coagulation cascades, and multiple virus- and bacterium-associated infection pathways (Figure 4C). The concordance between WGCNA and RNA-seq analyses indicates that NDV infection is accompanied by coordinated activation of immune- and stress-related signaling pathways together with suppression of metabolic and biosynthetic programs in HCT116 cells.

### 3.5. Genome-Wide CRISPR Screening Identifies Host Determinants of NDV-Mediated Cytotoxicity in HCT116 Cells

Based on the integrated analyses of WGCNA and RNA-seq, we identified several candidate pathways potentially involved in regulating NDV-mediated tumor cell killing. To identify host determinants of NDV susceptibility in an unbiased manner and without prior candidate-gene selection, a genome-wide CRISPR-Cas9 knockout screen was performed in HCT116 cells using the human GeCKO v2 lentiviral library, in which each gene is independently targeted by six distinct sgRNAs [25] (Figure 5A). This multi-sgRNA design improves the robustness of gene-level identification by minimizing the influence of differences in individual sgRNA editing efficiency and off-target activity. Following puromycin selection, a stable pooled knockout population was established and subsequently challenged with NDV (MOI = 5). Surviving cells were collected after viral infection for sgRNA abundance profiling by next-generation sequencing. In this screening strategy, positively selected genes represent genes whose knockout confers resistance to NDV-mediated killing, resulting in enrichment of the corresponding sgRNAs after viral challenge. Conversely, negatively selected genes represent genes whose knockout sensitizes cells to NDV-induced cell death, leading to depletion of their corresponding sgRNAs. Candidate genes were identified using the thresholds of *p* < 0.05 and |log_2_FC| > 1. To minimize potential biases caused by genes intrinsically involved in cell proliferation or survival, candidate genes identified from the screen were compared with previously annotated essential growth-regulatory genes present in the GeCKO library. Among the 462 positively selected genes identified in HCT116 cells, only three overlapped with the 1230 genes known to influence cellular proliferation, indicating minimal interference from baseline growth-associated effects and supporting the reliability of the screening results. After removing overlapping genes, a total of 459 positively selected genes and 513 negatively selected genes were retained for downstream analyses (Table S7).

GO enrichment analysis was subsequently performed (Table S8). Positive-selection genes were mainly enriched in steroid and purine metabolism, nuclear organization, MAPK cascade regulation, and growth-related processes, together with transcriptional and receptor-associated functions. In contrast, negative-selection genes were enriched in immune and inflammatory regulation, ribosome biogenesis, and intracellular signaling, including tumor necrosis factor production and immune response regulation. Collectively, the GO analysis suggests that positive- and negative-selection screens identify distinct host functional programs contributing to NDV susceptibility.

KEGG pathway enrichment analysis revealed (Table S9) that positively selected genes were predominantly associated with cellular metabolic processes, protein biosynthesis, and intracellular signaling pathways (Figure 5B). Representative pathways included retinol metabolism, tryptophan metabolism, terpenoid backbone biosynthesis, O-glycan biosynthesis, protein processing in the endoplasmic reticulum, and cGMP–PKG signaling. These findings suggest that genes supporting NDV-induced cytotoxicity are largely involved in maintaining metabolic activity and biosynthetic capacity, indicating that efficient viral oncolysis may depend on an intact host metabolic network. In contrast, negatively selected genes were enriched in pathways associated with cytokine signaling, innate immune responses, programmed cell death, and stress adaptation (Figure 5C). Significant pathways included cytokine–cytokine receptor interaction, Toll-like receptor signaling, NOD-like receptor signaling, chemokine signaling, apoptosis, necroptosis, and mitophagy. These results indicate that disruption of antiviral defense mechanisms and cell survival pathways enhances NDV-mediated cytotoxicity, highlighting the importance of host immune signaling in restricting viral oncolytic activity.

To further identify key regulatory nodes within the screening datasets, PPI networks were constructed using the positively and negatively selected genes. Network topology analysis identified *POLR2G* and *SREBF1* as central hubs in the positive-selection network, whereas *SRC* showed the highest connectivity in the negative-selection network (Figure 5D,E). Candidate prioritization, however, also considered concordance with the WGCNA modules and RNA-seq pathways. Because *SRC* showed limited agreement with these datasets, *TNFRSF1A*, *TNFAIP3*, and *CASP9* were selected based on their relatively high network rankings and functional links to innate immune signaling and programmed cell death. Overall, the integrated analyses indicated that positive-selection genes were associated mainly with metabolic and genetic information-processing pathways, whereas negative-selection genes were linked to antiviral defense and cell-death regulation. These findings suggest that NDV-mediated oncolysis depends on the balance between host metabolic support for viral replication and antiviral mechanisms that restrict cytotoxicity, and they nominate *POLR2G*, *SREBF1*, *TNFRSF1A*, *TNFAIP3*, and *CASP9* for subsequent functional validation.

### 3.6. Validation of POLR2G and SREBF1 as Positive Regulators of NDV-Mediated Oncolysis

To validate candidate genes identified through the integrated multi-omics analysis, *POLR2G* and *SREBF1* were selected for functional characterization. These candidates were prioritized due to their central positions in the PPI network (derived from the CRISPR positive-selection screen) and their involvement in pathways enriched in both the WGCNA positive regulatory module and the set of downregulated genes identified by RNA-seq. These overlapping pathways were primarily involved in cellular metabolism, transcriptional regulation, and DNA replication.

CRISPR/Cas9-mediated gene disruption was performed in HCT116 cells using sgRNAs targeting *POLR2G* and *SREBF1*. Following transfection, the cells were maintained for 72 h to allow sufficient expression of the delivered sgRNA constructs and the mCherry reporter, as well as adequate time for CRISPR-mediated genome editing. Seventy-two hours after transfection, approximately 70% of cells exhibited mCherry fluorescence, indicating efficient plasmid delivery, and mCherry-positive cells were subsequently enriched by fluorescence-activated cell sorting. Quantitative RT-PCR confirmed significant reductions in the mRNA expression levels of both genes compared with their corresponding control groups. Western blot analysis further verified efficient depletion of *POLR2G* and *SREBF1* proteins following gene knockout. Stable overexpression cell lines were also generated for both candidates; however, successful overexpression was achieved only for *POLR2G*, as confirmed by both qRT-PCR and Western blotting (Figure 6A–C).

To determine whether these genes influence NDV replication, viral genome copy numbers were quantified by absolute quantitative PCR following NDV infection. Modulation of *POLR2G* or *SREBF1* expression resulted in reproducible changes in viral genome abundance. Specifically, knockout of either gene reduced viral genome copy numbers, whereas POLR2G overexpression led to an increase in viral genome abundance. Notably, these changes were consistent with the corresponding trends in the cytotoxicity induced by NDV, suggesting that POLR2G and SREBF1 have a certain impact on viral replication (Figure 6E). Viral genome copies were measured at 36 hpi to allow sufficient viral replication and accumulation for reliable quantification.

The effects of these genes on NDV-mediated cytotoxicity were subsequently evaluated by LDH release assays at 24 and 36 hpi. Knockout of either POLR2G or SREBF1 significantly attenuated NDV-induced cytotoxicity compared with the corresponding control groups at both time points. In contrast, *POLR2G* overexpression significantly enhanced NDV-mediated cytotoxicity at both 24 h and 36 hpi (Figure 6D). Collectively, these findings identify *POLR2G* and *SREBF1* as positive regulators of NDV-mediated oncolysis. Although modulation of these genes resulted in changes in viral genome abundance, their effects on NDV-induced cytotoxicity were substantially more pronounced, suggesting that these host factors not only alter viral replication but also enhance the susceptibility of HCT116 cells to NDV-mediated killing.

### 3.7. Genetic and Pharmacological Validation of Host-Restrictive Pathways Regulating NDV Replication and Oncolysis

To validate the host-restrictive factors identified by the CRISPR/Cas9 negative-selection screen and integrated multi-omics analysis, *TNFRSF1A*, *TNFAIP3*, and *CASP9* were selected for functional characterization because they represent key components of the TLR4, NF-κB, JAK–STAT, and apoptotic signaling pathways. CRISPR/Cas9-mediated gene knockout was performed in HCT116 cells, and efficient depletion of each target gene was confirmed by quantitative RT-PCR and Western blot analysis (Figure 7A–C).

To determine the functional consequences of gene disruption on NDV infection, viral genome copy numbers were quantified at 36 hpi. Knockout of TNFRSF1A, TNFAIP3, or CASP9 resulted in increased viral genome abundance compared with the corresponding control cells, indicating that disruption of these host factors promoted NDV replication (Figure 7F). Consistent with the increase in viral replication, LDH release assays showed that NDV-induced cytotoxicity was comparable among the different groups at 24 hpi but became significantly enhanced at 36 h following knockout of TNFRSF1A, TNFAIP3, or CASP9, with CASP9 deficiency producing the greatest increase in cell death (Figure 7D). These findings suggest that loss of these host factors facilitates NDV replication and consequently enhances NDV-mediated oncolysis.

To further validate the signaling pathways identified by the integrated multi-omics analyses, HCT116 cells were treated with pharmacological inhibitors targeting representative components of the JAK–STAT, TLR4/NF-κB, and apoptotic pathways before NDV infection. Inhibition of JAK/TLR4 signaling with Ruxolitinib and Resatorvid significantly enhanced NDV-mediated cytotoxicity, whereas inhibition of NF-κB signaling with BAY 11-7082 had no significant effect on NDV-induced cytotoxicity. Treatment with Z-LEHD-FMK promoted NDV-mediated cytotoxicity, while inhibition of apoptosis with Z-VAD-FMK suppressed NDV-induced cell killing (Figure 7E). Measurement of viral genome copy numbers demonstrated changes that were generally consistent with the corresponding alterations in cytotoxicity (Figure 7G), indicating that pharmacological modulation of these signaling pathways influences NDV oncolytic activity, through regulation of viral replication. Specifically, Ruxolitinib and Z-LEHD-FMK increased NDV-induced cell mortality, whereas the effect of Resatorvid was more pronounced at 36 hpi than at 24 hpi. BAY 11-7082 did not significantly alter cell mortality, while Z-VAD-FMK markedly reduced NDV-induced mortality at both time points. These cytotoxicity patterns were broadly paralleled by changes in viral genome copy numbers, which generally increased following treatments that enhanced cell mortality but decreased following Z-VAD-FMK treatment.

Collectively, both genetic and pharmacological approaches consistently demonstrated that host signaling pathways identified by the integrated multi-omics analyses regulate NDV infection. Disruption of TNFRSF1A, TNFAIP3, and CASP9, or pharmacological modulation of the JAK–STAT, TLR4, NF-κB, and apoptotic pathways, altered viral replication and was accompanied by corresponding changes in NDV-mediated cytotoxicity. These findings support a model in which host antiviral and inflammatory signaling pathways limit NDV replication, thereby restricting the efficiency of NDV-mediated tumor cytotoxicity.

## 4. Discussion

Oncolytic Newcastle disease virus (NDV) has attracted considerable attention as a promising anticancer therapeutic owing to its ability to selectively lyse tumor cells while simultaneously stimulating antitumor immune responses [27]. However, substantial heterogeneity in tumor susceptibility and host antiviral defense mechanisms remains a major obstacle limiting its therapeutic efficacy. Identifying the host determinants that regulate NDV-mediated cytotoxicity is therefore essential for improving virotherapy and developing rational combination strategies.

In the present study, we integrated WGCNA, RNA sequencing, and genome-wide CRISPR-Cas9 screening to systematically investigate the host regulatory model governing NDV-mediated oncolysis. Unlike previous studies that primarily focused on individual genes or signaling pathways, our integrative strategy revealed two coordinated yet opposing host regulatory programs that collectively determine the response of colorectal cancer cells to NDV infection. One program was characterized by transcriptional and metabolic activities that positively correlated with NDV-induced cytotoxicity, whereas the other consisted predominantly of antiviral and inflammatory signaling pathways that restricted viral killing. The convergence of three independent omics approaches provides strong evidence that these two host programs represent fundamental determinants of NDV susceptibility.

The metabolism-associated permissive program emerged as one of the most consistent findings across all datasets. WGCNA identified gene modules positively associated with NDV-mediated cytotoxicity that were enriched in ribosome biogenesis, RNA polymerase, mitochondrial function, oxidative phosphorylation, DNA replication, and AMPK/mTOR signaling. Similarly, genes identified by CRISPR positive-selection screening were predominantly involved in transcription, protein synthesis, and cellular metabolism. Previous studies have demonstrated that NDV infection extensively remodels host metabolism to support efficient viral infection, including alterations in glycolysis, mitochondrial function, and lipid metabolism [28]. Consistent with these observations, our multi-omics analyses suggest that an active metabolic state is closely associated with increased susceptibility to NDV-induced cytotoxicity.

Functional validation further supported this conclusion. *POLR2G* and *SREBF1* were prioritized as representative candidates based on the integrated results of the genome-wide CRISPR-Cas9 screen, transcriptomic analysis, and regulatory network analysis, as well as their potential roles in the metabolic regulation of NDV-mediated oncolysis. *POLR2G*, an essential subunit of RNA polymerase II, and *SREBF1*, a master regulator of lipid and cholesterol biosynthesis, were selected as representative genes involved in this bidirectional regulation of host metabolism [29,30]. Knockout of either gene significantly attenuated NDV-induced cytotoxicity, whereas POLR2G overexpression further enhanced virus-mediated cell death. Notably, these genetic manipulations were accompanied by corresponding changes in viral genomic copy numbers, indicating that the altered cytotoxicity was partially attributable to changes in viral replication. These findings suggest that *POLR2G* and *SREBF1* may influence NDV-induced oncolysis, by modulating the cellular metabolic environment that supports viral replication. Previous findings showed that NDV-induced, ubiquitin–proteasome-dependent degradation of FLCN promotes *TFE3/TFEB* nuclear translocation and suppresses *SREBF1c*-dependent de novo fatty acid synthesis [28]. We therefore hypothesize that *SREBF1* acts as a downstream metabolic component of this FLCN-regulated pathway, potentially facilitating viral replication by reducing the energetic burden of fatty acid synthesis. Collectively, these results suggest that host metabolic activity is not merely a prerequisite for viral biosynthesis but also an important determinant of whether infected tumor cells ultimately undergo efficient virus-induced cytotoxicity.

The second regulatory program identified in this study consisted predominantly of host antiviral and inflammatory signaling pathways. Both WGCNA and RNA-seq analyses demonstrated significant enrichment of JAK-STAT, TLR4, NF-κB, cytokine signaling, and innate immune-related pathways among negatively correlated or infection-induced gene sets. These findings are consistent with the established antiviral response initiated by NDV, in which viral RNA is recognized by pattern recognition receptors such as RIG-I and MDA5, resulting in activation of IRF3, NF-κB, and JAK-STAT signaling and subsequent induction of interferon-stimulated genes (ISGs) [31,32,33,34]. While these antiviral responses are indispensable for host defense, they may also limit the therapeutic efficacy of oncolytic viruses by establishing an antiviral cellular state.

The functional relevance of this antiviral regulatory pattern was further supported by both genetic and pharmacological validation. Knockout of *TNFRSF1A*, *TNFAIP3*, and *CASP9* altered NDV-mediated cytotoxicity, highlighting the contribution of inflammatory and cell death signaling to the host response against NDV infection. Consistent with these genetic findings, pharmacological inhibition of JAK signaling by Ruxolitinib significantly enhanced NDV-induced cytotoxicity. Inhibition of TLR4 and CASP9 also increased NDV-induced cell death, further indicating that multiple host signaling pathways contribute to the regulation of NDV-mediated oncolysis. Notably, changes in cytotoxicity were variably associated with changes in viral genome abundance, suggesting that enhanced viral replication contributes to the increased cell death phenotype in some contexts, whereas additional replication-independent mechanisms may also participate in pathway-specific regulation of NDV-induced oncolysis. In contrast, inhibition of NF-κB produced no appreciable effect on NDV-induced cytotoxicity. This lack of effect may reflect the context-dependent and bidirectional functions of NF-κB signaling, which can exert both antiviral and pro-survival or pro-death effects depending on the cellular and infectious context [35]; thus, its net contribution may be limited in the HCT116-NDV model. Interestingly, although CASP9 inhibition enhanced NDV-induced cytotoxicity, broad inhibition of apoptosis/caspase activity did not produce a similar phenotype. This apparent discrepancy may be explained by the broader activity of the pan-caspase inhibitor, which suppresses multiple caspases and potentially other downstream cell-death execution pathways, thereby reducing overall NDV-induced cell death and masking the effect observed with selective CASP9 inhibition. Collectively, these findings indicate that the regulation of NDV-mediated oncolysis involves multiple interconnected antiviral, inflammatory, and cell death pathways, with their effects on cytotoxicity arising from both replication-dependent and replication-independent mechanisms. These findings are consistent with previous studies showing that inhibition of interferon signaling can enhance the efficacy of several RNA oncolytic viruses and further support the targeting of specific host regulatory pathways as a potential strategy for improving NDV-based virotherapy [36,37]. A model of host cellular processes associated with oncolytic activity is presented in Figure 8.

Several limitations should be acknowledged. First, mechanistic validation was performed primarily in HCT116 cells, and additional colorectal cancer models, particularly models with distinct genetic and metabolic backgrounds, as well as in vivo studies, will be required to determine the generalizability and therapeutic relevance of these findings. Second, viral infection was assessed mainly by viral genome quantification. Measurements of infectious viral titers, interferon production, and interferon-stimulated gene expression would provide a more comprehensive assessment of how the identified host factors influence different stages of the NDV life cycle and antiviral responses. Third, although POLR2G, SREBF1, TNFRSF1A, TNFAIP3, CASP9, and JAK–STAT signaling were identified as representative regulatory nodes, the molecular connections between host metabolism, inflammatory and antiviral signaling, viral replication, and NDV-induced cell death remain incompletely defined. In particular, whether these factors act independently or converge on common cellular processes that determine NDV permissiveness warrants further investigation. Given the essential physiological functions of some candidate genes, complete systemic inhibition may not be clinically feasible; partial, transient, or tumor-targeted modulation may therefore represent more appropriate therapeutic strategies. Future studies using xenograft models established with gene-knockout or gene-overexpression tumor cells, together with genetically engineered colorectal cancer models, are needed to validate the biological and therapeutic relevance of these targets in vivo.

In conclusion, this study sufficiently characterized the host regulatory network controlling NDV-mediated oncolysis through integrative multi-omics analyses and functional validation. Our findings reveal a bidirectional regulatory framework, in which host metabolic and biosynthetic activity promotes NDV-mediated tumor cell killing, whereas antiviral and inflammatory defense programs restrict viral propagation and oncolytic efficacy. *POLR2G* and *SREBF1* were identified as representative pro-oncolytic determinants, while *TNFRSF1A*, *TNFAIP3*, and *CASP9* emerged as anti-oncolytic host factors. Furthermore, the JAK–STAT and TLR4 pathways were identified as a key and pharmacologically actionable antiviral barrier, as their inhibition enhanced NDV-mediated cytotoxicity. Collectively, these findings advance our understanding of NDV–host interactions and provide a conceptual and experimental foundation for host-directed combination strategies designed to enhance the therapeutic efficacy of NDV-based oncolytic virotherapy.

## Figures and Tables

**Figure 1:**
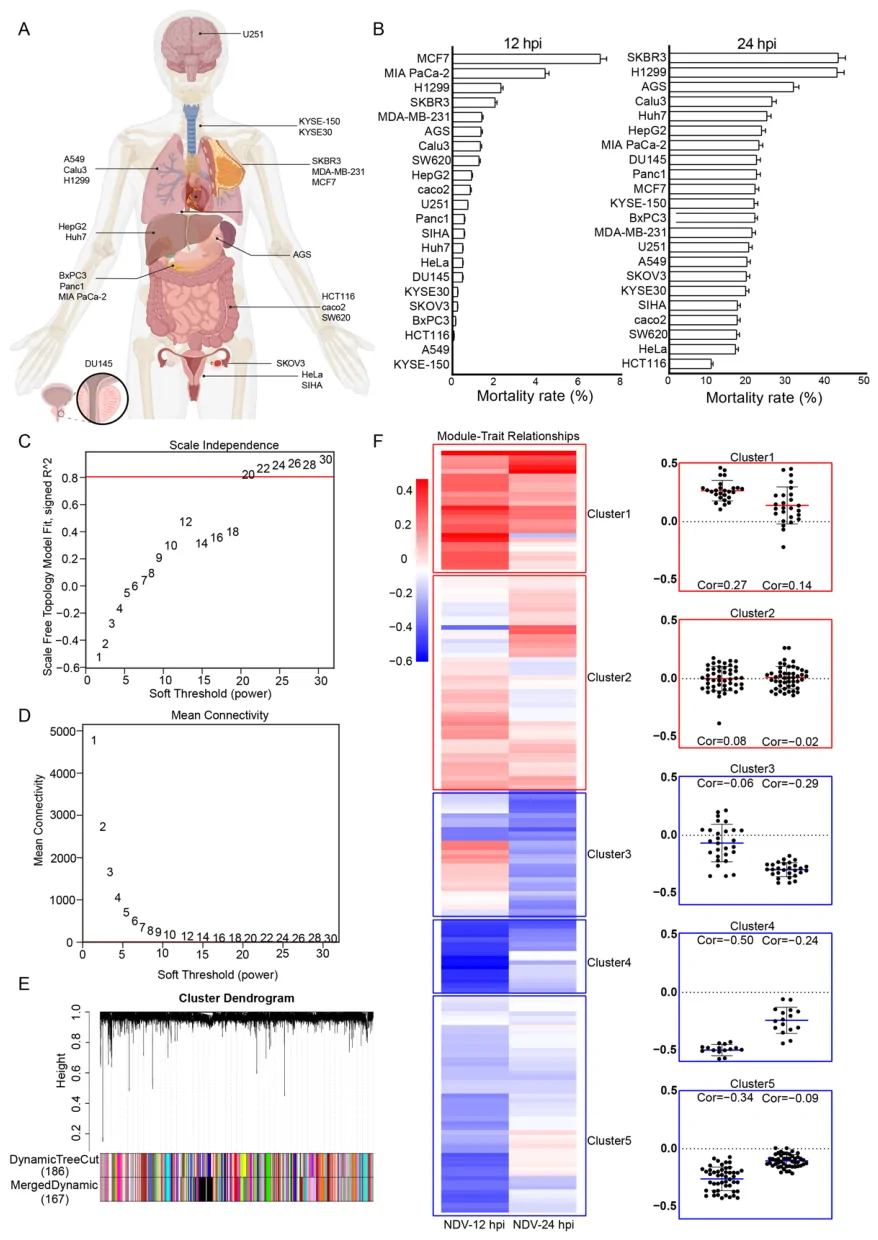
Identification of gene co-expression modules associated with NDV susceptibility in human cancer cell lines. (**A**) Schematic illustration showing the tissue origins of the 22 human cancer cell lines derived from 11 different cancer types included in this study. This figure includes elements created with BioRender.com under a valid publication license “created in BioRender. Feng, S. (2026). https://BioRender.com/on9r1x5”. (**B**) Susceptibility of different cancer cell lines to NDV-mediated oncolysis. Cells were infected with the NDV LaSota strain (MOI = 5), and cytotoxicity was quantified by LDH release assays at 12 and 24 hpi (*n* = 8). Cell lines were ranked according to their mortality rates. (**C**,**D**) Selection of the soft-thresholding power for WGCNA. The scale-free topology fit index (**C**) and mean connectivity (**D**) were calculated under different soft-thresholding powers. A power of 20 was selected to generate a scale-free co-expression network. (**E**) Hierarchical clustering dendrogram of expressed genes. Gene modules were identified using the dynamic tree-cut algorithm and merged according to module eigengene similarity. Each color represents an individual co-expression module. (**F**) Association between co-expression modules and NDV susceptibility. Left, expression patterns of module eigengenes across samples. Right, correlations between module eigengenes and NDV-induced cytotoxicity. The red module showed a significant positive correlation with NDV-mediated cytotoxicity, whereas the blue module displayed a significant negative correlation.

**Figure 2:**
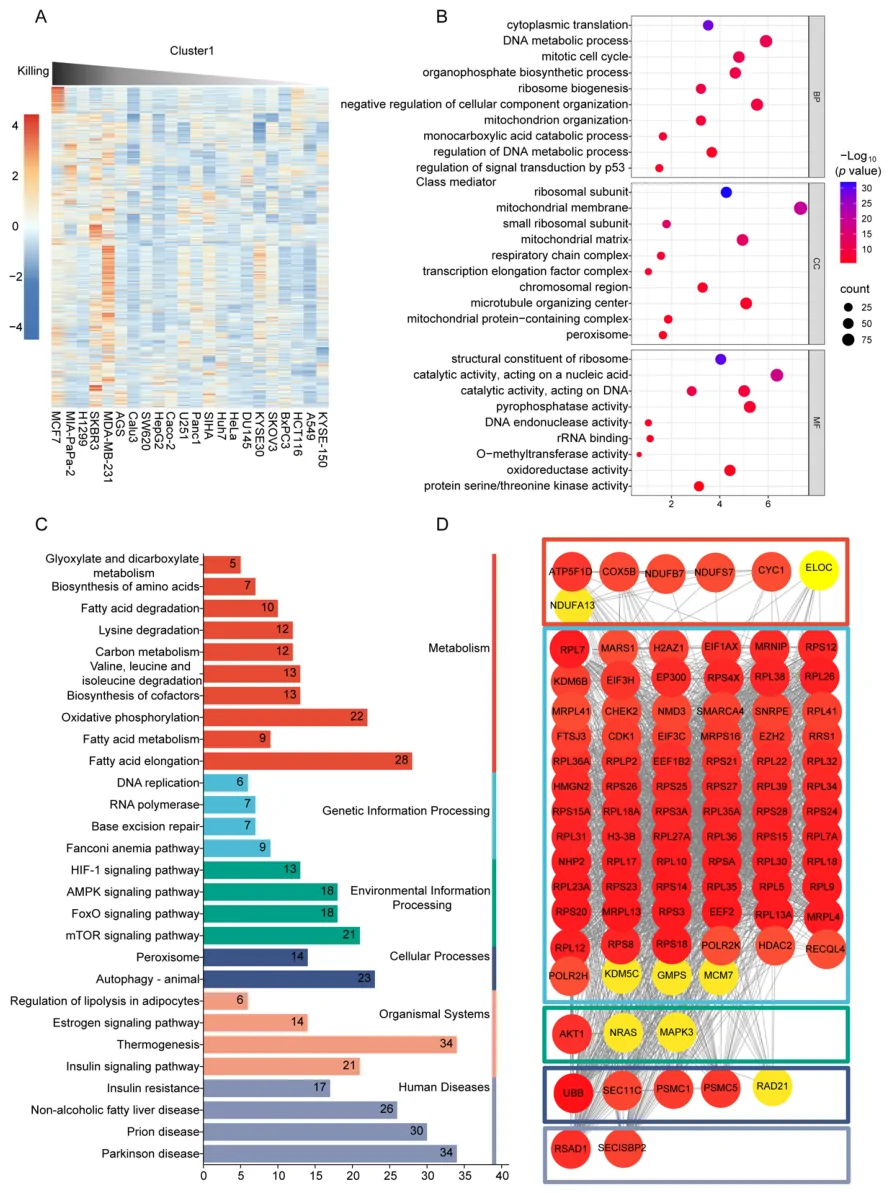
Functional enrichment and protein–protein interaction network analysis of the positively correlated WGCNA module associated with NDV-mediated oncolysis. Both 12- and 24-h cytotoxicity measurements were included as phenotypic traits in the WGCNA. Because module membership was determined from transcriptomic expression data and did not vary according to the phenotypic time point, the same module gene set was used for the subsequent GO/KEGG enrichment and PPI analyses. (**A**) Heatmap showing the expression profiles of the 1380 genes in the positively correlated WGCNA module across the 22 human cancer cell lines. Cell lines are arranged according to descending susceptibility to NDV-induced cytotoxicity. Red and blue indicate relatively high and low gene expression levels, respectively. (**B**) GO enrichment analysis of genes in the positively correlated module. The top enriched BP, CC and MF terms are shown. Dot size represents the number of enriched genes, and dot color indicates the enrichment significance (−log_10_ *p* value). (**C**) KEGG pathway enrichment analysis of the positively correlated module. Genes were predominantly enriched in metabolic pathways, including amino acid biosynthesis, oxidative phosphorylation, fatty acid degradation, and cofactor biosynthesis, as well as pathways involved in genetic information processing, DNA replication and repair, AMPK/mTOR signaling, autophagy, and human diseases. (**D**) PPI network of the positively correlated module constructed using the STRING database and visualized with Cytoscape. Node color reflects the relative degree of connectivity within the network. Highly connected hub genes were mainly involved in metabolic regulation, transcriptional regulation, DNA replication and repair, and mitochondrial function, suggesting that maintenance of cellular metabolism and biosynthetic capacity is closely associated with efficient NDV-mediated oncolysis.

**Figure 3:**
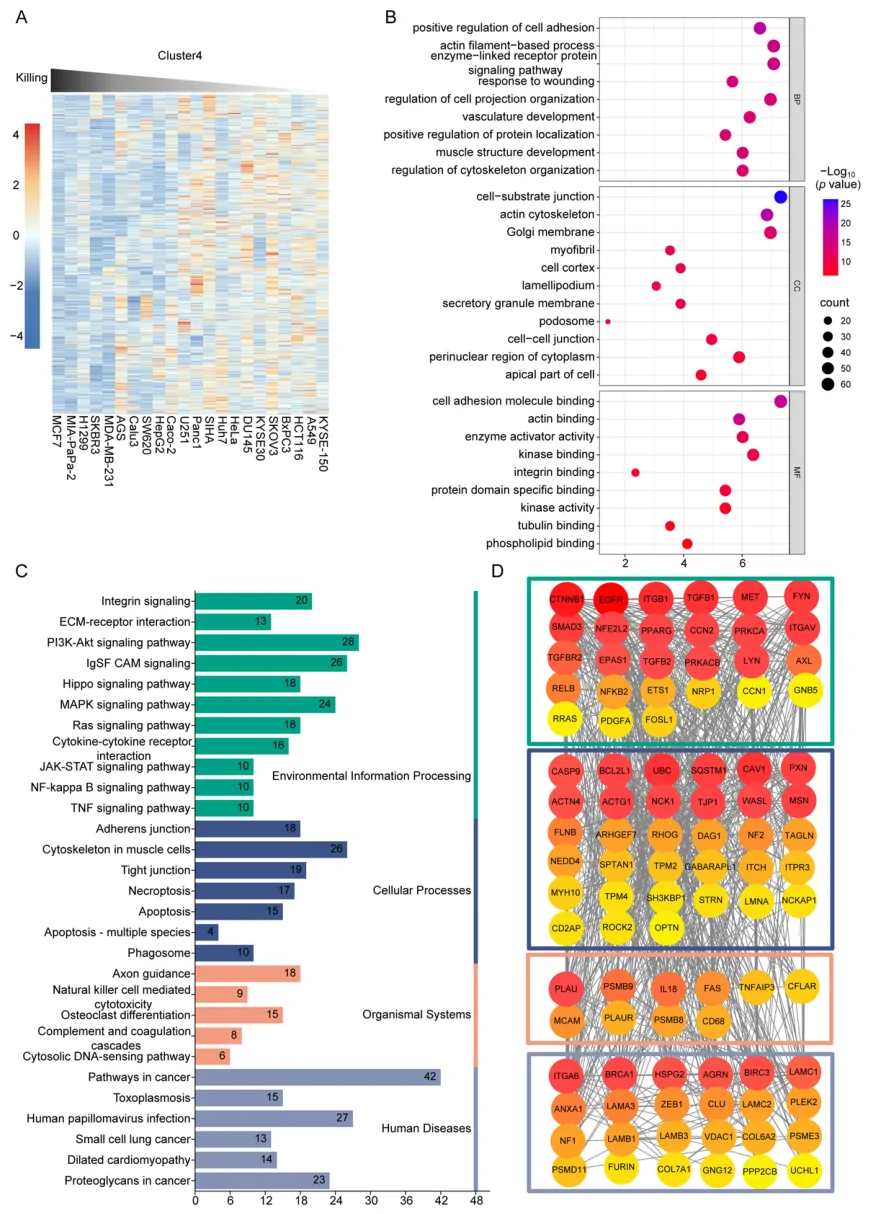
Functional enrichment and protein–protein interaction network analysis of the negatively correlated WGCNA module associated with NDV-mediated oncolysis. (**A**) Heatmap showing the expression profiles of the 853 genes in the negatively correlated WGCNA module across the 22 human cancer cell lines. Cell lines are arranged according to descending susceptibility to NDV-induced cytotoxicity. Red and blue indicate relatively high and low gene expression levels, respectively. (**B**) GO enrichment analysis of the negatively correlated module. BP, CC and MF terms are presented. Dot size represents the number of enriched genes, whereas dot color indicates enrichment significance (−log_10_ *p* value). (**C**) KEGG pathway enrichment analysis of the negatively correlated module. The enriched pathways were primarily associated with cytokine-mediated signaling, cell adhesion, actin cytoskeleton organization, JAK–STAT, TNF, NF-κB, PI3K–Akt, apoptosis, necroptosis, phagosome, and pathogen infection pathways, highlighting the involvement of antiviral innate immunity, inflammatory signaling, and cellular defense mechanisms. (**D**) PPI network of the negatively correlated module. Highly connected hub genes were enriched in pathways regulating inflammatory signaling, innate antiviral responses, cytoskeletal remodeling, and programmed cell death, providing candidate therapeutic targets for enhancing NDV-mediated oncolytic efficacy through modulation of host antiviral signaling.

**Figure 4:**
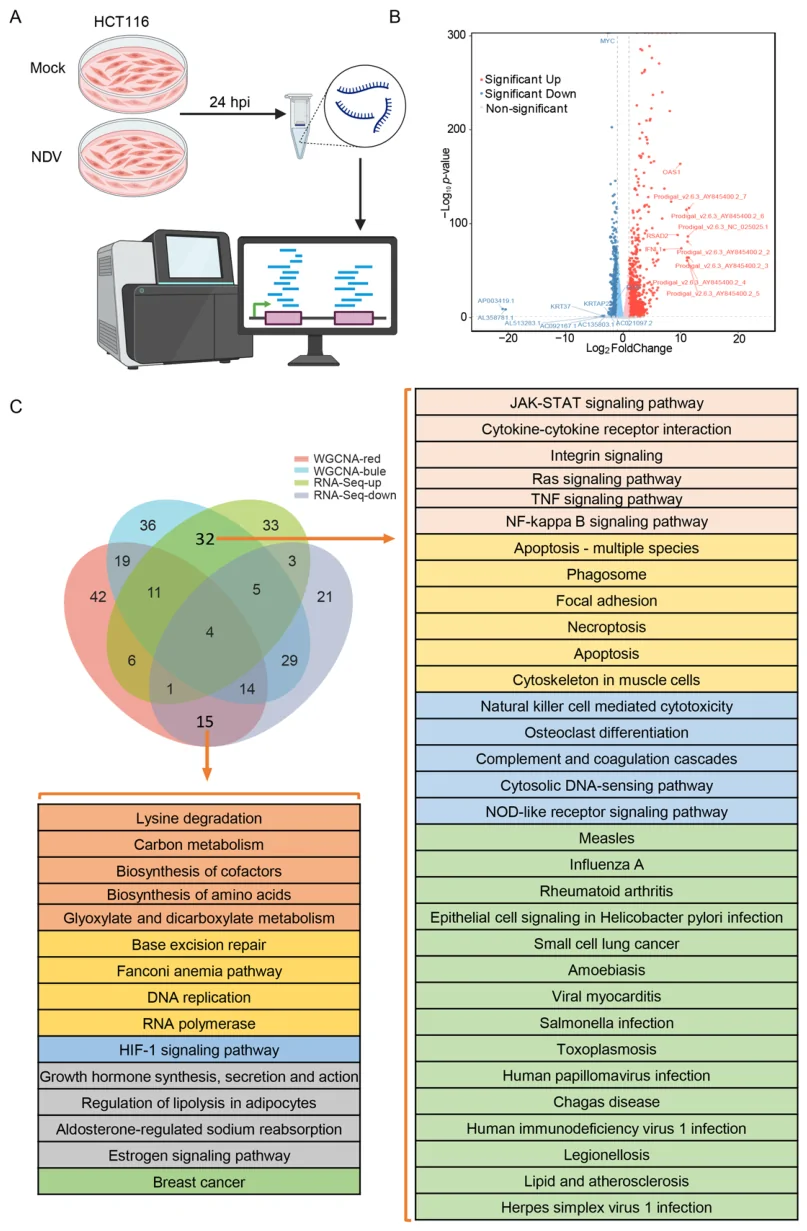
Integrative analysis of WGCNA and RNA-seq identifies host pathways associated with NDV resistance in HCT116 cells. (**A**) Experimental workflow for transcriptome profiling. HCT116 cells were mock-treated or infected with NDV (LaSota strain, MOI = 5) for 24 h before RNA extraction and RNA sequencing. (**B**) Differential gene expression analysis of NDV-infected versus mock-treated HCT116 cells. Differentially expressed genes (DEGs) were identified using the criteria of |log_2_FC| > 1 and *p* < 0.05, yielding 2117 upregulated and 752 downregulated genes. (**C**) Integration of WGCNA and RNA-seq datasets through KEGG pathway overlap analysis. KEGG pathways enriched in the positively correlated WGCNA module (WGCNA-red) were intersected with pathways enriched among downregulated DEGs, whereas pathways enriched in the negatively correlated WGCNA module (WGCNA-bule) were intersected with those enriched among upregulated DEGs. The intersecting pathways suggest that suppression of metabolic and biosynthetic pathways, together with activation of antiviral innate immune responses, inflammatory signaling, and programmed cell death-associated pathways, represents the major molecular features associated with NDV resistance in HCT116 cells and provided the basis for subsequent CRISPR screening and functional validation.

**Figure 5:**
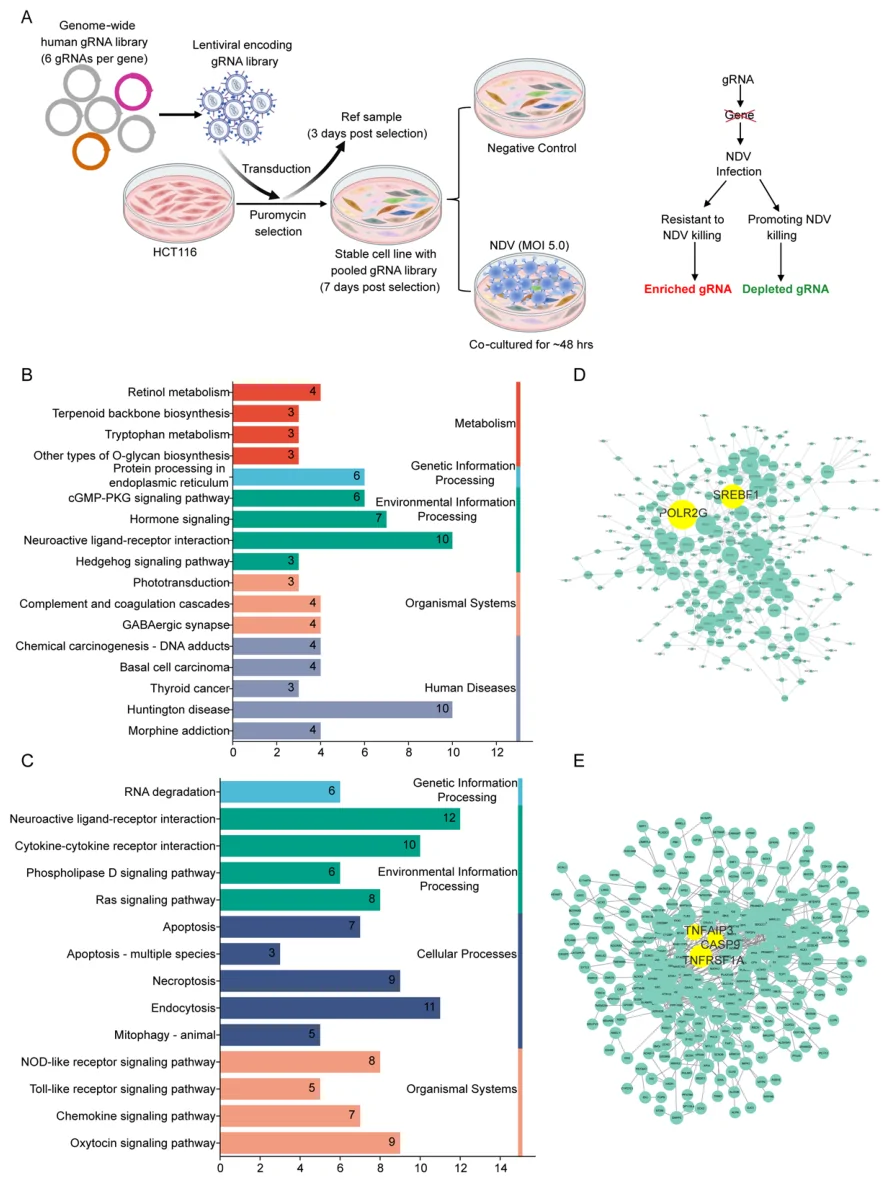
Genome-wide CRISPR/Cas9 screening identifies host factors regulating NDV-mediated cytotoxicity in HCT116 cells. (**A**) Schematic overview of the genome-wide CRISPR/Cas9 loss-of-function screen. HCT116 cells were transduced with a lentiviral human genome-wide sgRNA library containing six sgRNAs per gene, followed by puromycin selection to establish a pooled knockout cell population. sgRNAs enriched after NDV infection represent genes whose loss confers resistance to NDV-mediated killing (positive selection), whereas depleted sgRNAs indicate genes whose loss enhances NDV-mediated cytotoxicity (negative selection). (**B**) KEGG pathway enrichment analysis of positively selected genes identified from the CRISPR screen, representing host factors whose knockout confers resistance to NDV-mediated killing. (**C**) KEGG pathway enrichment analysis of negatively selected genes identified from the CRISPR screen, representing host factors whose knockout sensitizes HCT116 cells to NDV-mediated killing. (**D**) PPI network of positively selected genes. Hub genes were identified based on network topology analysis, with representative candidate genes highlighted. (**E**) PPI network of negatively selected genes. Representative hub genes associated with enhanced NDV-mediated cytotoxicity are highlighted. The size of nodes in the network therefore represents gene connectivity (degree).

**Figure 6:**
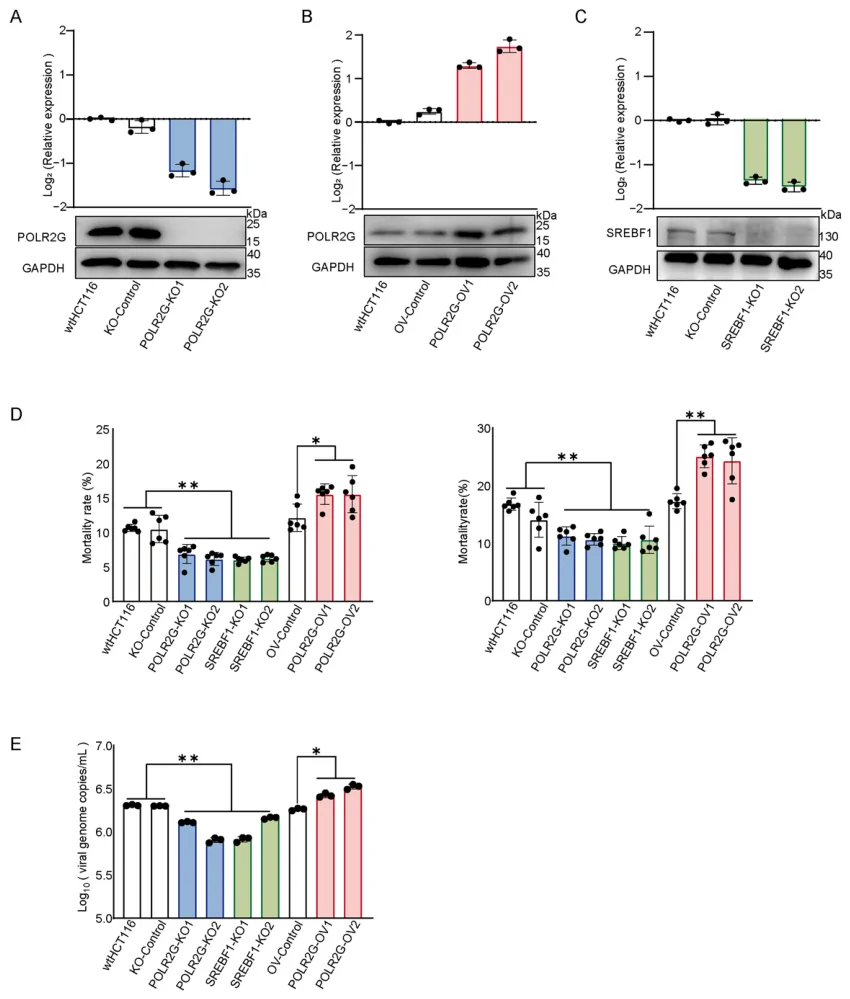
Validation of candidate host factors regulating NDV-mediated cytotoxicity in HCT116 cells. (**A**) Validation of *POLR2G* knockout (KO) cell lines. Relative *POLR2G* mRNA expression was determined by quantitative real-time PCR (qRT-PCR), and protein expression was confirmed by Western blot analysis. GAPDH was used as the loading control. (**B**) Validation of *POLR2G* overexpression (OV) cell lines. Relative *POLR2G* mRNA expression was measured by qRT-PCR, and protein expression was verified by Western blot analysis. GAPDH served as the loading control. (**C**) Validation of *SREBF1* knockout (KO) cell lines. Relative *SREBF1* mRNA expression was determined by qRT-PCR, and protein expression was confirmed by Western blot analysis. GAPDH was used as the loading control. Data in A–C are presented as log_2_ (relative expression) expression levels relative to the control group. (**D**) Effects of *POLR2G* and *SREBF1* knockout or overexpression on NDV-induced cytotoxicity in HCT116 cells. Wild-type (WT), corresponding control (KO-Control and OV-Control), gene-knockout, and gene-overexpression cells were infected with NDV. Cell death was assessed by an LDH release assay at 24 and 36 h post-infection (hpi) (*n* = 6) and is presented as the percentage of cell mortality. (**E**) Viral replication in wild-type and genetically modified HCT116 cells following NDV infection. Viral genome copy numbers were quantified by absolute quantitative PCR at 36 hpi (*n* = 3) and are presented as log_10_ viral genome copies per milliliter (log_10_ viral genome copies/mL). Data are presented as mean ± SD from three independent biological experiments. Statistical analyses were performed using one-way ANOVA followed by Dunnett’s multiple-comparison test. Statistical significance is indicated as *p* < 0.05 (*) and *p* < 0.01 (**).

**Figure 7:**
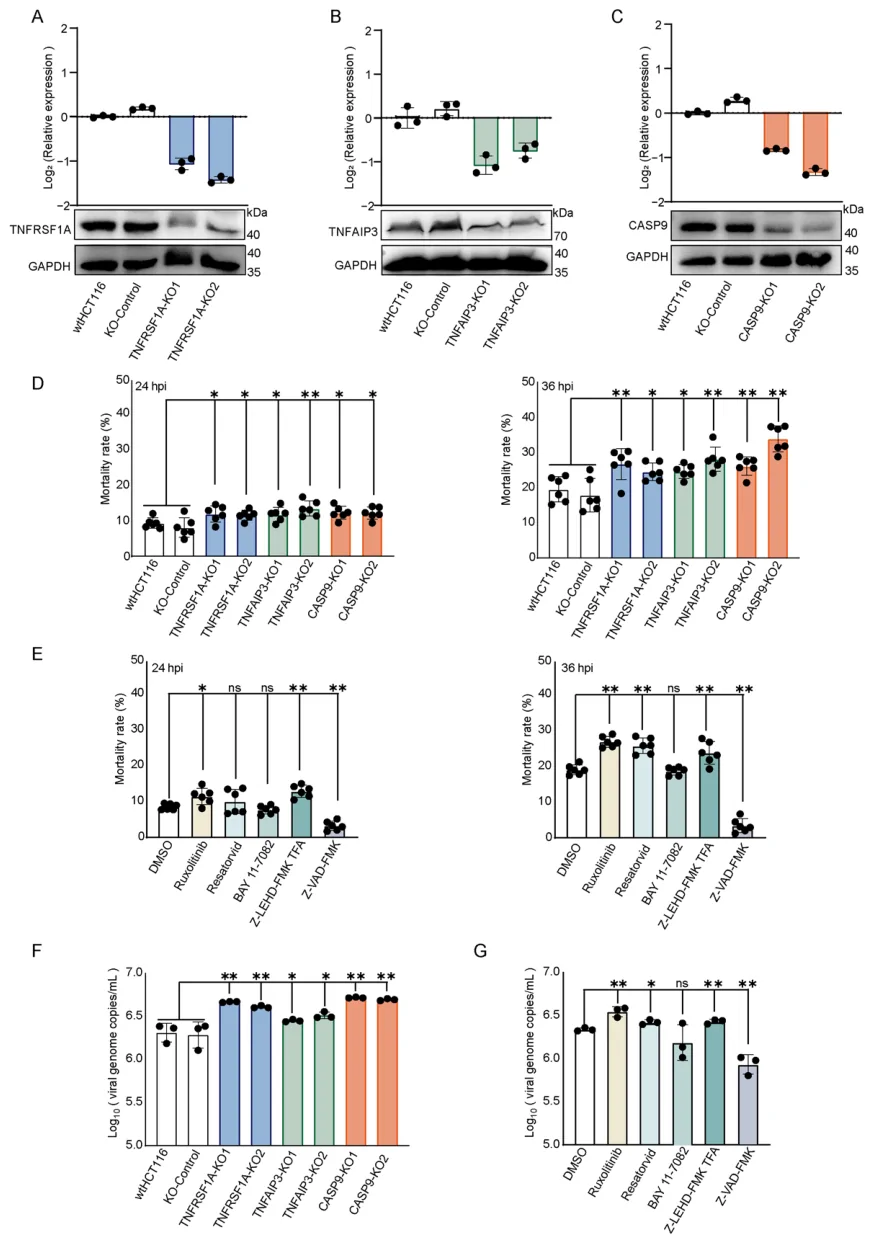
Genome-wide screening identifies host antiviral and cell death pathways regulating NDV-induced oncolysis. KO1 and KO2 represent two independently generated knockout cell lines established using two distinct sgRNAs targeting different sites within the corresponding gene. (**A**–**C**) Validation of *TNFRSF1A*, *TNFAIP3* and *CASP9* knockout (KO) cell lines. Relative *TNFRSF1A*, *TNFAIP3* and *CASP9* mRNA expression was determined by qRT-PCR, and protein expression was confirmed by Western blot analysis. GAPDH was used as the loading control. (**D**) Effects of *TNFRSF1A*, *TNFAIP3*, and *CASP9* knockout on NDV-induced cytotoxicity. Wild-type, KO-control, and independent knockout cell lines were infected with NDV, and cell death was quantified by LDH release assay at 24 and 36 hpi (*n* = 6). Data are presented as the percentage of cell mortality. (**E**) HCT116 cells were pretreated for 6 h with the indicated concentrations of the JAK1/2 inhibitor Ruxolitinib (10 μM), TLR4 inhibitor Resatorvid (0.5 μM), NF-κB inhibitor BAY 11-7082 (1 μM), caspase-9 inhibitor Z-LEHD-FMK TFA (1 μM), or pan-caspase inhibitor Z-VAD-FMK (10 μM), followed by NDV infection. NDV-induced cytotoxicity was quantified by LDH release assays at 24 and 36 hpi (*n* = 6) and is presented as the mortality rate. (**F**,**G**) Viral replication in wild-type, KO-control, and TNFRSF1A-, TNFAIP3-, or CASP9-knockout HCT116 cells following NDV infection. Effects of the indicated inhibitors on NDV replication. Viral genome copy numbers were quantified by absolute quantitative PCR at 36 hpi (*n* = 3) and are presented as log10 viral genome copies per milliliter (log_10_ viral genome copies/mL). Data are presented as mean ± SD from three independent biological experiments. Statistical analyses were performed using one-way ANOVA followed by Dunnett’s multiple-comparison test. Statistical significance is indicated as *p* < 0.05 (*) and *p* < 0.01 (**).

**Figure 8:**
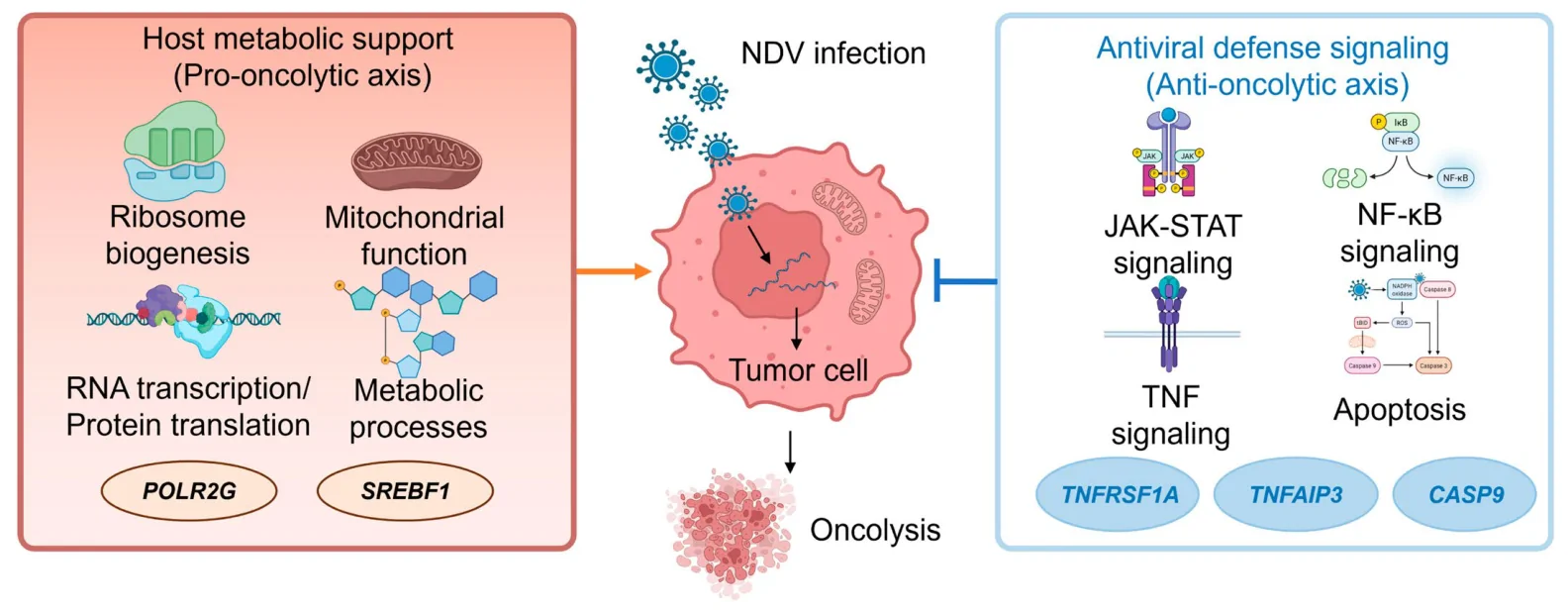
Model of host cellular processes associated with oncolytic activity. The model depicts two opposing functional axes in tumor cells. The host metabolic support axis includes ribosome biogenesis, mitochondrial function, RNA transcription, protein translation, and metabolic processes, with *POLR2G* and *SREBF1* highlighted as candidate associated genes. The antiviral defense axis encompasses JAK–STAT, NF-κB, and TNF signaling, together with apoptosis-related processes, with *TNFRSF1A*, *TNFAIP3*, and *CASP9* highlighted as candidate associated genes. The orange arrow and blue inhibitory bar indicate the proposed supportive and restrictive effects on oncolytic activity, respectively.

**Table 1:** Experimental models: Cell lines.

Cell Lines	Origin Source	Source	Identifier
U251	Brain	Yaji Biotechnology	YS301C
KYSE-150	Esophagus	Yaji Biotechnology	YS168C
KYSE30	Esophagus	Yaji Biotechnology	YS321C
SKBR3	Breast	CTCC	GDC0286
MDA-MB-231	Breast	ATCC	HTB-26™
MCF7	Breast	ATCC	HTB-22™
A549	Lung	ATCC	CCL-185™
Calu3	Lung	CTCC	GDC0647
H1299	Lung	CTCC	GDC0640
HepG2	Liver	CTCC	GDC0690
Huh7	Liver	CTCC	GDC0134
AGS	Stomach	CTCC	GDC0247
BxPC3	Pancreas	CTCC	GDC0663
Panc1	Pancreas	CTCC	GDC0309
MIA PaCa-2	Pancreas	CTCC	GDC0255
HCT116	Colon	ATCC	CCL-247EMT™
Caco2	Colon	CTCC	GDC0153
SW620	Colon	Yaji Biotechnology	YS285C
SKOV3	Ovary	CTCC	GDC0114
HeLa	Cervix	ATCC	CRM-CCL-2™
SIHA	Cervix	CTCC	GDC0110
DU145	Prostate	CTCC	GDC0704
293T	Embryonic kidney	ATCC	CRL-3216™

**Table 2:** sgRNA used for plasmid construction.

sgRNA Name	Sequence (5′-3′)
*POLR2G*-sgRNA1	CTGCTGCACCCGCGCTACTT
*POLR2G*-sgRNA2	GTTGGACTCTTCACAGAAAT
*SREBF1*-sgRNA1	GCTGGGCGAGCCGTGCGATC
*SREBF1*-sgRNA2	GCGCTGCTGACCGACATCGA
*TNFRSF1A*-sgRNA1	CAGGGGTTATTGGACTGGTC
*TNFRSF1A*-sgRNA2	CAGCTGCTCCAAATGCCGAA
*TNFAIP3*-sgRNA1	CGGGGCTTTGCTATGATACT
*TNFAIP3*-sgRNA2	TACAGATACCCCATTGTTCT
*CASP9*-sgRNA1	CTTCGTTTCTGCGAACTAAC
*CASP9*-sgRNA2	CGGTGCTCTGGACTGCTGCG

**Table 3:** Primers used for RT-qPCR.

Primer Name	Sequence (5′-3′)
*POLR2G*-F	CAAGGTTGGACTCTTCACAGAAAT
*POLR2G*-R	GTAATCGTCCATCGGGAGCCAAT
*SREBF1*-F	TGCTGACCGACATCGA
*SREBF1*-R	TCAAATAGGCCAGGGAAGT
*TNFRSF1A*-F	CAGGGGTTATTGGACTG
*TNFRSF1A*-R	CATTTGGAGCAGCTG
*TNFAIP3*-F	GCTTTGCTATGATACTCG
*TNFAIP3*-R	GTATCTGTAGCATTC
*CASP9*-F	CTGCATTTCATGGTGGAGGTG
*CASP9*-R	ACAGCCGTGAGAGAGAATG
*GAPDH*-F	GTCTCCTCTGACTTCAACAGCG
*GAPDH*-R	ACCACCCTGTTGCTGTAGCCAA

## Data Availability

The original data presented in the study are openly available in NCBI at https://www.ncbi.nlm.nih.gov/geo/query/acc.cgi?acc=GSE297244 (accessed on 24 August 2026) or accession number GSE297244.

## Supplementary Materials

The following supporting information can be downloaded at: https://www.mdpi.com/article/10.3390/microorganisms14102156/s1, Table S1: Significant genes identified by WGCNA; Table S2: GO analysis of significant genes identified by WGCNA; Table S3: KEGG pathway analysis of significant genes identified by WGCNA; Table S4: Differentially expressed genes (DEGs) identified by RNA-sequencing; Table S5: GO analysis of DEGs identified by RNA-sequencing; Table S6: KEGG pathway analysis of DEGs identified by RNA-sequencing; Table S7: Significant genes identified by genome-wide CRISPR-based screen; Table S8: GO analysis of significant genes identified by genome-wide CRISPR-based screen; Table S9: KEGG pathway analysis of significant genes identified by genome-wide CRISPR-based screen.

## References

[1] Balch C.M., Liao N., Lam D.S.C., Weitzel J.N., Xu R.H., Attard G., Bunn P.A., Eggermont A.M.M., He J., Kitagawa Y. (2026). The global cancer crisis: A review of growing burden, deepening inequality and initiatives for prevention and early detection. Ecancermedicalscience.

[2] Bray F., Laversanne M., Sung H., Ferlay J., Siegel R.L., Soerjomataram I., Jemal A. (2024). Global cancer statistics 2022: GLOBOCAN estimates of incidence and mortality worldwide for 36 cancers in 185 countries. CA Cancer J. Clin..

[3] Wang Y., Yu F., Qiu F., Fang K. (2025). Global burden of respiratory system cancers in 2022 and 2050: Incidence and mortality estimates from GLOBOCAN. BMC Cancer.

[4] Kazaryan A.M., Marangos I.P., Røsok B.I., Rosseland A.R., Villanger O., Fosse E., Mathisen O., Edwin B. (2010). Laparoscopic resection of colorectal liver metastases: Surgical and long-term oncologic outcome. Ann. Surg..

[5] Kripp M., Wieneke J., Kienle P., Welzel G., Brade J., Horisberger K., Wenz F., Post S., Gencer D., Hofmann W.K. (2012). Intensified neoadjuvant chemoradiotherapy in locally advanced rectal cancer–impact on long-term quality of life. Eur. J. Surg. Oncol..

[6] Sforza V., Martinelli E., Ciardiello F., Gambardella V., Napolitano S., Martini G., Della Corte C., Cardone C., Ferrara M.L., Reginelli A. (2016). Mechanisms of resistance to anti-epidermal growth factor receptor inhibitors in metastatic colorectal cancer. World J. Gastroenterol..

[7] Kwong T.T., Xiong Z., Zhang Y., Wu H., Cao J., Pak-Chun Wong P., Liu X., Wang J., Wong C.H., Man-Kit Tse G. (2025). Overcoming immunotherapy resistance in hepatocellular carcinoma by targeting myeloid IL-8/CXCR2 signaling. Mol. Ther..

[8] Abushukair H., Ababneh O., Zaitoun S., Saeed A. (2022). Primary and secondary immune checkpoint inhibitors resistance in colorectal cancer: Key mechanisms and ways to overcome resistance. Cancer Treat. Res. Commun..

[9] Andtbacka R.H., Kaufman H.L., Collichio F., Amatruda T., Senzer N., Chesney J., Delman K.A., Spitler L.E., Puzanov I., Agarwala S.S. (2015). Talimogene Laherparepvec Improves Durable Response Rate in Patients With Advanced Melanoma. J. Clin. Oncol..

[10] Sun H., Chen X., Wang Y. (2025). Strategies for the modification of vaccinia virus towards a better vaccine vector. Zoonoses.

[11] Tan E.W., Abd-Aziz N., Poh C.L., Tan K.O. (2022). Engineered Oncolytic Adenoviruses: An Emerging Approach for Cancer Therapy. Pathogens.

[12] Kanai R., Zaupa C., Sgubin D., Antoszczyk S.J., Martuza R.L., Wakimoto H., Rabkin S.D. (2012). Effect of γ34.5 deletions on oncolytic herpes simplex virus activity in brain tumors. J. Virol..

[13] Shalhout S.Z., Miller D.M., Emerick K.S., Kaufman H.L. (2023). Therapy with oncolytic viruses: Progress and challenges. Nat. Rev. Clin. Oncol..

[14] Salameh H., Naseem N., Chattha M.A., Ramesh J., Ramy H., Cizkova D., Kubatka P., Büsselberg D. (2025). Oncolytic Virotherapy in Colorectal Cancer: Mechanistic Insights, Enhancer Strategies, and Translational Combinations. Cells.

[15] Alwithenani A., Hengswat P., Chiocca E.A. (2025). Oncolytic viruses as cancer therapeutics: From mechanistic insights to clinical translation. Mol. Ther..

[16] Zhang Y., Feng S., Yi G., Jin S., Zhu Y., Liu X., Zhou J., Li H. (2025). Genome-Wide Screening Reveals the Oncolytic Mechanism of Newcastle Disease Virus in a Human Colonic Carcinoma Cell Line. Viruses.

[17] Zhang X., He L., Zhang R. (2024). Enhanced expression of two ISGs as potential biomarkers of prognosis in HIV-infected immunological non-responders. Zoonoses.

[18] Wang M., Jiang K., Aicher A., Heeschen C. (2025). Engineering the tumor microenvironment: Oncolytic NDV to facilitate CAR-T cell therapy. J. Transl. Med..

[19] Mayasich S., Schumann P., Peter G. (2024). In silico analysis of cross-species sequence variability in host interferon antiviral pathway proteins and SARS-CoV-2 susceptibility. Zoonoses.

[20] Zhong L., Gan L., Wang B., Wu T., Yao F., Gong W., Peng H., Deng Z., Xiao G., Liu X. (2025). Hyperacute rejection-engineered oncolytic virus for interventional clinical trial in refractory cancer patients. Cell.

[21] Abedizadeh R., Majidi F., Khorasani H.R., Abedi H., Sabour D. (2024). Colorectal cancer: A comprehensive review of carcinogenesis, diagnosis, and novel strategies for classified treatments. Cancer Metastasis Rev..

[22] Qu Y., Wang S., Jiang H., Liao Y., Qiu X., Tan L., Song C., Nair V., Yang Z., Sun Y. (2024). Newcastle disease virus infection induces parthanatos in tumor cells via calcium waves. PLoS Pathog..

[23] Pérez-Domínguez F., Quezada-Monrás C., Cárcamo L., Muñoz J.P., Carrillo-Beltrán D. (2025). Oncolytic Viruses as a Novel Therapeutic Approach for Colorectal Cancer: Mechanisms, Current Advances, and Future Directions. Cancers.

[24] Alanazi A., Ibrahim M.N., El Azab E.F., Elithy M.A. (2026). Host Cell Virus Interactions: Molecular Mechanisms, Immune Modulation, Viral Pathogenesis, and Emerging Therapeutic Targets. Viruses.

[25] Sanjana N.E., Shalem O., Zhang F. (2014). Improved vectors and genome-wide libraries for CRISPR screening. Nat. Methods.

[26] Wu D., Smyth G.K. (2012). Camera: A competitive gene set test accounting for inter-gene correlation. Nucleic Acids Res..

[27] Vijayakumar G., McCroskery S., Palese P. (2020). Engineering Newcastle Disease Virus as an Oncolytic Vector for Intratumoral Delivery of Immune Checkpoint Inhibitors and Immunocytokines. J. Virol..

[28] Yang M., Kan X., Li X., Sun Y., Liao Y., Qiu X., Tan L., Song C., Ding C. (2025). Newcastle disease virus induces degradation of folliculin to regulate host cell energy metabolism and facilitate viral replication. Poult. Sci..

[29] Schier A.C., Taatjes D.J. (2020). Structure and mechanism of the RNA polymerase II transcription machinery. Genes Dev..

[30] Wei G., Huang Y., Li W., Xie Y., Zhang D., Niu Y., Zhao Y. (2025). SREBF1-based metabolic reprogramming in prostate cancer promotes tumor ferroptosis resistance. Cell Death Discov..

[31] Sun Y., Ding N., Ding S.S., Yu S., Meng C., Chen H., Qiu X., Zhang S., Yu Y., Zhan Y. (2013). Goose RIG-I functions in innate immunity against Newcastle disease virus infections. Mol. Immunol..

[32] Dias Junior A.G., Sampaio N.G., Rehwinkel J. (2019). A Balancing Act: MDA5 in Antiviral Immunity and Autoinflammation. Trends Microbiol..

[33] Popli S., Chakravarty S., Fan S., Glanz A., Aras S., Nagy L.E., Sen G.C., Chakravarti R., Chattopadhyay S. (2022). IRF3 inhibits nuclear translocation of NF-κB to prevent viral inflammation. Proc. Natl. Acad. Sci. USA.

[34] Wu Y.Y., Wu F.H., Tseng C.Y., Chiang P.K., Muniri M., Liu H.J. (2026). The interplay between oncolytic viruses, Toll-like receptor signaling, and chemotherapy in overcoming tumor immune tolerance. Front. Cell Infect. Microbiol..

[35] Struzik J., Szulc-Dąbrowska L. (2018). NF-κB Signaling in Targeting Tumor Cells by Oncolytic Viruses-Therapeutic Perspectives. Cancers.

[36] Zhu Y., Xu M., Li M. (2026). Propranolol enhances the oncolytic effect of newcastle disease virus on canine mammary tumor cell by modulating the IFN-I-mediated JAK-STAT signaling pathway. Vet. Q..

[37] Kumar P., Kumar A., Kumar A., Kumar H. (2026). Essential role of hsa-miR-203a-3p in type I interferons immune homeostasis during influenza and NDV infection. RNA Biol..

